# Treatment with placental growth factor attenuates myocardial ischemia/reperfusion injury

**DOI:** 10.1371/journal.pone.0202772

**Published:** 2018-09-13

**Authors:** Yabing Zhang, Chang Cao, Juan Xin, Peilin Lv, Dongxu Chen, Shiyue Li, Hui Yang, Chan Chen, Bin Liu, Qian Li

**Affiliations:** 1 Department of Anesthesiology, West China Hospital of Sichuan University, Chengdu, Sichuan, China; 2 Department of Burn and Plastic Surgery, West China Hospital of Sichuan University, Chengdu, Sichuan, China; Indiana University School of Medicine, UNITED STATES

## Abstract

Studies have established that oxidative stress plays an important role in the pathology of myocardial ischemia/reperfusion injury (MIRI). Vascular endothelial growth factor receptor 1 (VEGFR1) activation was reported to reduce oxidative stress and apoptosis. In the present study, we tested the hypothesis that the activation of VEGFR1 by placental growth factor (PlGF) could reduce MIRI by regulating oxidative stress. Mouse hearts and neonatal mouse cardiomyocytes were subjected to ischemia/reperfusion (I/R) and oxygen glucose deprivation (OGD), respectively. PlGF pretreatment markedly ameliorated I/R injury, as demonstrated by reduced infarct size and improved cardiac function. The protection was associated with a reduction of cardiomyocyte apoptosis. Similarly, our in vitro study showed that PlGF treatment improved cell viability and reduced cardiomyocyte apoptosis. Also, activation of VEGFR1 by PlGF suppressed intracellular and mitochondrial reactive oxygen species (ROS) generation. However, VEGFR1 neutralizing monoclonal antibody, which preventing PlGF binding, totally blocked this protective effect. In conclusion, activation of VEGFR1 could protect heart from I/R injury by suppression of oxidative stress and apoptosis.

## Introduction

Coronary heart disease is the leading cause of morbidity and mortality in the world.[1] After an acute myocardial infarction (AMI), the most effective strategy for reducing the size of a myocardial infarct and improving the clinical outcome is early and successful myocardial reperfusion. The reperfusion may, however, result in paradoxical cardiomyocyte dysfunction and worsen tissue damage, in a process known as “myocardial ischemia/reperfusion injury” (MIRI).[2–4]

Oxidative stress caused by elevated levels of reactive oxygen species (ROS) or reactive nitrogen species (RNS) can lead to protein, lipid, and DNA damage, and rapidly proceed to irreversible cell death by apoptosis and necrosis.[5–7] Presence of ROS in excess of the antioxidant capacity of the heart is one of the main mechanisms underlying the pathology of MIRI.[4, 8] As such, the oxidative stress is considered to be of paramount importance for MIRI development.

Placental growth factor (PlGF), a selective ligand of VEGFR1 (Flt-1), is a member of the PDGF/VEGF family growth factors. It is mainly expressed in placenta, heart, and lungs tissue.[9] The role of VEGFR1 in the heart is not clear. Previous studies have shown that the activation of VEGFR1, similar to VEGFR2, was responsible for angiogenesis, vasculogenesis, and endothelial cell growth, but without the associated side-effects, such as edema, hypotension, and hemangioma-genesis.[10–12]

A study demonstrated that exogenous administration of PlGF could reduce infarct size and improve cardiac function following AMI, by enhancing angiogenesis and arteriogenesis. Co-administration of soluble VEGFR1 inhibited the beneficial effects from PlGF.[13] However, it has been reported that PlGF-VEGFR1 signaling plays an important role in decreasing apoptosis of tumor cells, which set VEGFR1 apart from VEGFR2.[14] Studies suggested that VEGF-VEGFR1 signal transduction pathway mediated the cardioprotection of ischemic preconditioning. In the heterozygous VEGFR1 knockout mice, cardioprotection of ischemic preconditioning was not as effective as found in wild type counterparts.[6, 15, 16] It demonstrated that stimulation of VEGFR1 leaded to the anti-apoptotic and non-angiogenic properties in MIRI and the importance in cardioprotection.

Little is known about the role of VEGFR1 in MIRI. In present study, we hypothesized that PlGF-VEGFR1 signaling represents a protective pathway in the heart and attenuates MIRI. We investigated the effect of exogenous PlGF protein on cardiac function and prognosis after myocardial ischemia/reperfusion (I/R) and determined the role of PlGF-VEGFR1 in oxidative stress of cardiomyocyte induced by hypoxia/reoxygenation (H/R).

## Materials and methods

### Experimental animals

All the experiments were performed in adherence with the Guide for the Care and Use of Laboratory Animals published by the US National Institutes of Health and approved by the Institutional Animal Experimental Ethics Committee of Sichuan University. C57BL/6J mice (aged 8–10 weeks) were used and all the animals were housed in cages (two or three mice per cage) on a 12/12 h light-dark cycle with free access to food and water.

### Myocardial ischemia/reperfusion protocol

The myocardial I/R surgery was performed as previously described.[17] Briefly, the mice were anesthetized with intraperitoneal injection of ketamine (120 mg/kg) and xylazine (4 mg/kg), intubated, and ventilated. The left intercostal thoracotomy was performed and the left anterior descending coronary artery (LAD) was ligated with a 7–0 silk suture under a surgical microscope. Following 45 minutes of LAD occlusion, the LAD ligature was released, and the myocardium was reperfused for 24 h (for infarct size and cardiac function assays). The mice in PlGF and MF-1 groups were pretreated with exogenous PlGF (R&D Systems) (0.05μg/g per day, i.v., for 2 days) and neutralizing monoclonal Flt-1 antibody (MF-1) (R&D Systems) (1mg, i.p., 2 days before surgery) respectively, followed by 45 min ischemia and 24 h reperfusion. Sham-operated control mice underwent the same procedure, but the suture under the LAD remained untied.

### Determination of cardiac function and myocardial infarct size

At the end of reperfusion, mice were anesthetized, and cardiac function was determined with a 12-MHz transducer (i13L, Vivi7 Dimension, GE) in the left lateral position. After assessment, infarct size was determined by Evans blue/triphenyltetrazolium chloride (TTC) staining as previously described.[18]

### Histology

At the end of the experiment, the hearts were removed, fixed by 10% formalin and embedded with paraffin, sliced into pieces of 5 μm sections. Paraffin sections were stained with hematoxylin and eosin. Images were captured using a microscope.

### Measurement of cytokines and cardiac enzymes

After reperfusion, TNF-α, IL-1β and IL-6 levels in myocardial tissue homogenate, as well as the serum level of creatine kinase-MB (CK-MB) and troponin T (cTnT) were determined using mouse ELISA kits according to the manufacturer’s instructions.

### Western blot analysis

Cardiac tissue homogenate proteins from the left ventricular tissue were separated by SDS-PAGE gels and transferred to nitrocellulose membranes. Standard Western blots analysis were performed using antibodies against p-VEGFR1 (R&D Systems, AF4170), VEGFR1 (Abcam, ab32152), p-AKT (Abcam, ab81283), AKT (Abcam, ab32505), p-GSK3β (Abcam, ab75745), GSK3β (Abcam, ab32391), p-FOXO3a (Abcam, ab47285), FOXO3a (Abcam, ab109629) and Cleaved Caspase-3 (CST, 9664s). Nitrocellulose membranes were then incubated with HRP-conjugated IgG antibody. Blotting was analyzed using ImageJ software.

### Determination of myocardial apoptosis

Paraffin sections of the left ventricular tissue were stained using a terminal deoxynucleotidyl transferase dUTP nick end labeling (TUNEL) apoptosis assay kit (Roche Ltd., Switzerland) following the manufacturer's instructions. Cells were defined as apoptotic if the entire nuclear area of the cell was positively labeled. The apoptotic index (AI) was calculated as the percentage of positively stained cells.

### Cardiomyocyte isolation and oxygen glucose deprivation

Primary cultured neonatal mouse cardiac cells were subjected to 6h oxygen glucose deprivation (OGD) and 4h reoxygenation. Mouse neonatal cardiomyocytes were isolated and cultured as previously described.[19] Cardiomyocytes were transferred into serum-free medium containing vehicle (sterile water) (Control and OGD groups), PlGF (100ng/mL) (OGD+PlGF group), or MF-1 (15μg/ml)+PlGF (100ng/mL) (OGD+PLGF+MF-1 group) and cultured overnight in a cell incubator prior to hypoxia/reoxygenation. For the cells of the OGD+PLGF+MF-1 group, administration of MF-1 was 2h earlier than that of PlGF. The cells were subjected to hypoxia followed by reoxygenation as previously described with minor modifications.[20] The cells of OGD, PlGF and OGD+PLGF+ MF-1 groups were incubated in Dulbecco's Modified Eagle Medium (DMEM) with no serum or glucose for 6h in a hypoxic chamber saturated with a 0.1% O_2_, 5% CO_2_, 95% N_2_ gaseous mix, humidified, and warmed at 37°C, for 6 h. Afterwards, the cells were reoxygenated for 4 h by incubation in normoxic conditions in glucose-containing, serum-free DMEM. In the Control group, the cardiomyocytes were maintained for 10 h by incubation in normoxic conditions in glucose-containing, serum-free DMEM. The experiments were repeated for four times.

### Determination of cell viability

At the end of reoxygenation, the cell viability was determined using cell counting kit-8 (CCK-8, Dojindo, Kumamoto, Japan).

### Determination of apoptosis by flow cytometry

Cell apoptosis was assessed using the Annexin V-FITC/PI Kit (Dojindo, Kumamoto, Japan) according to the manufacturer’s instruction. Briefly, the cells were harvested and resuspended in 500 μl of medium buffer after reoxygenation, mixed with FITC-labeled annexin V (5μl), and incubated for 15 minutes at room temperature. Finally, PI (5μl) was added, and the cells were evaluated by flow cytometry (Beckman, USA).

### Determination of ROS

Intracellular ROS was measured by MitoSOX (Thermo Fisher Scientific; Rockford, IL, USA) and 2’,7’-dichlorofluorescein diacetate (DCF) (Sigma, St Louis, MO, USA) as previously described.[21] At the end of reoxygenation, the cardiomyocytes were stained with 5 μM MitoSOX Red or 5 μM DCF to detect superoxide anion. Cells were washed and imaged using a Nikon-Eclipse80i confocal microscope with 561 nm and 488 nm excitation for MitoSOX Red and DCF respectively. The fluorescence intensity of MitoSOX Red and DCF were qualified with ImageJ software, as reported previously.[22] The data are presented as fold change in the median intensity of the fluorescence when compared with the respective controls.

## Results

### PlGF reduced myocardial infarction and improved cardiac function after I/R

**Fig 1:**
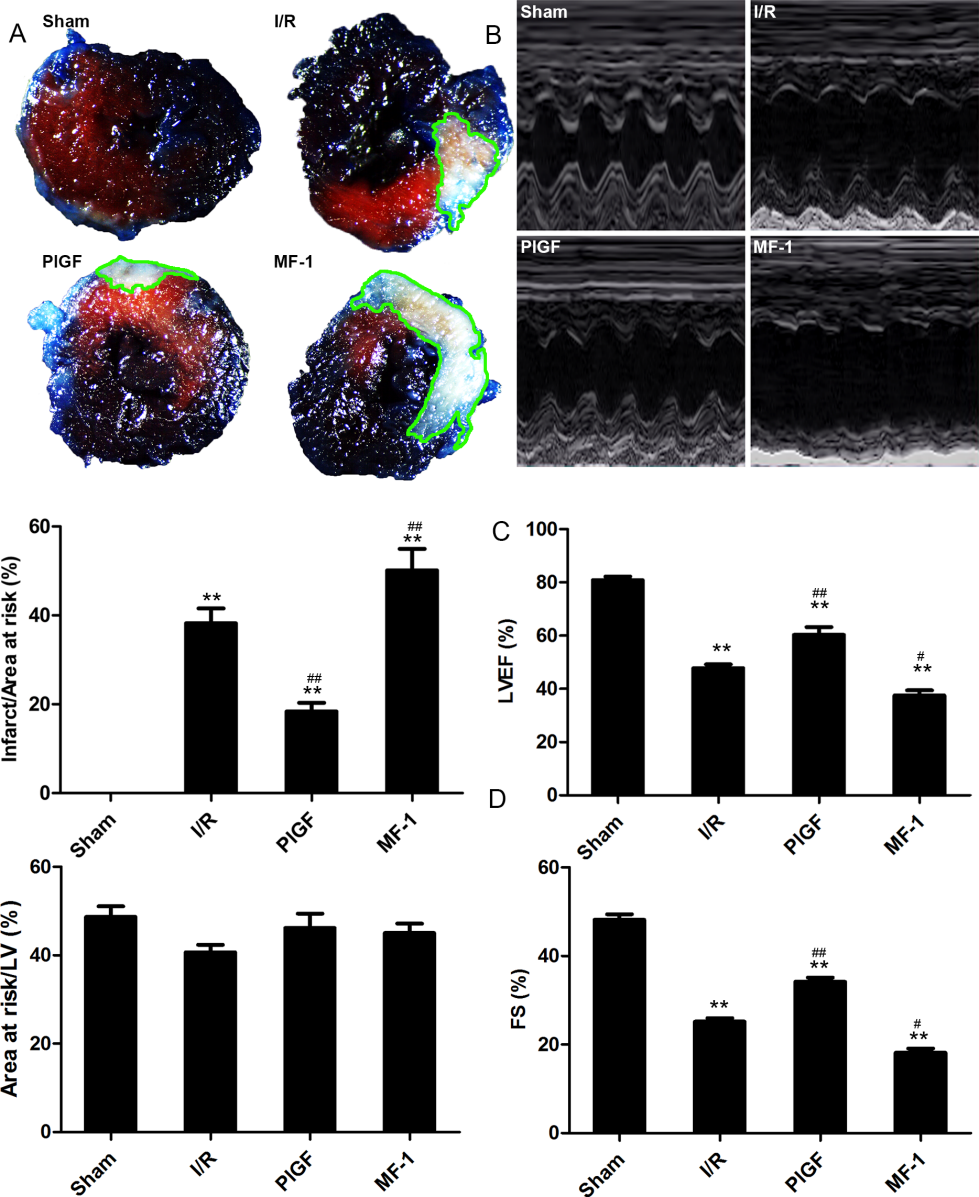
The effect of PlGF on myocardial infarction and improves cardiac function after I/R. (A) Representative images of the myocardial infarct size from different groups at 1 d after reperfusion. (B) Representative M-mode echocardiography images of different groups 1 d after reperfusion. (C) Ejection fraction and (D) fractional shortening (n = 6). Data were expressed as mean ± SEM. ** *P*<0.01 vs. the Sham group, ## *P*<0.01 vs. the I/R group.

I/R induced a significant infarction area. Mice treated with PlGF had significantly smaller infarct size, compared with mice in the I/R group (18% vs. 38%, P<0.01). MF-1 induced larger infarct size compared with the I/R group (51% vs. 38.2% P<0.01) (Fig 1A). To determine the effect of PlGF on cardiac function after I/R, twenty-four hours after reperfusion, we measured ejection fraction (EF) and fractional shortening (FS) by echocardiography. The EF and FS of I/R group decreased to 48% and 25% from baseline, respectively (P<0.01). Treatment with PlGF increased EF (60%) and FS (34%) after reperfusion, indicating improved cardiac function. The mice in the MF-1 group had significantly augmented left ventricle (LV) contractile function as demonstrated by EF (37.5%) and FS (18%), compared with the I/R group (P<0.05). (Fig 1B, 1C and 1D)

### PlGF suppressed cardiac histopathological changes and enzyme release

**Fig 2:**
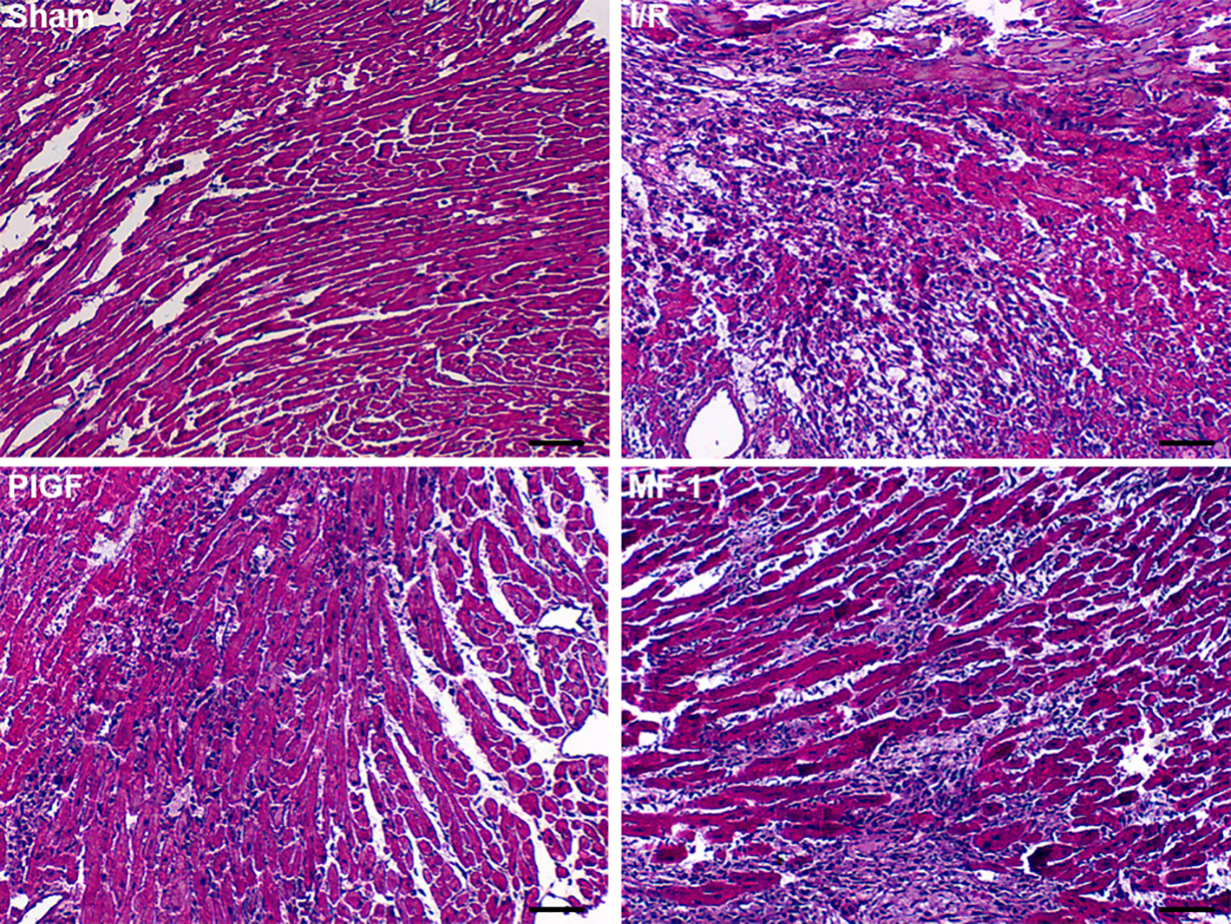
Effects of PlGF on myocardial histology after I/R injury. Relieved myocardium injury was presented in the hearts of PlGF group, compared with that of I/R group. (×100, scar bars: 50 μm).

**Fig 3:**
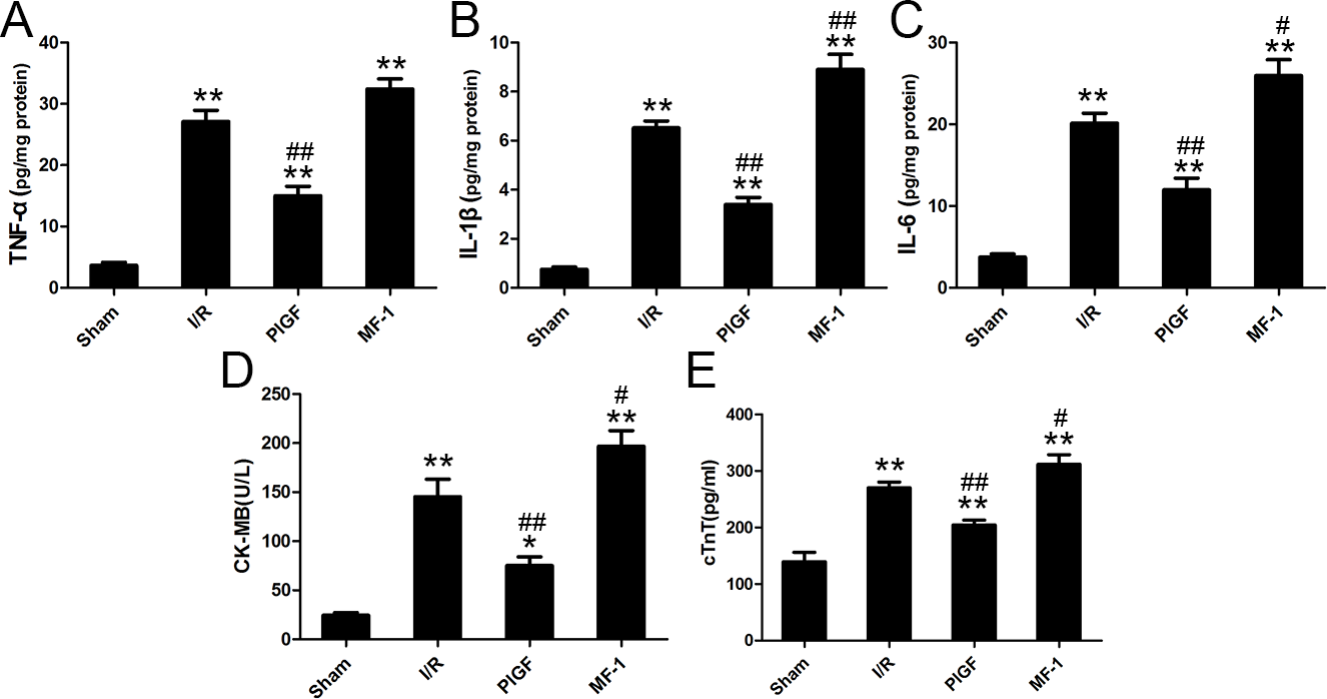
The effect of PlGF treatment on inflammatory cytokines and cardiac enzymes in serum after I/R. (A-C) The expression level of TNF-α, IL-1β and IL-6 after I/R (n = 6). (D) and (E) Total CK-MB and cTnT levels in serum (n = 6) Data were expressed as mean ± SEM. ** *P*<0.01 vs. the Sham group, # *P*<0.05 vs. the I/R group, ## *P*<0.01 vs. the I/R group.

HE staining showed that I/R resulted in aberrant myocardial fibers disordered transverse striation with marked inflammatory cell infiltration, which was prevented by PlGF treatment (Fig 2). After reperfusion, cytokine proteins including TNF-α, IL-1β and IL-6 were significantly upregulated. The levels of these cytokine proteins were significantly lower in the PlGF group as compared to the I/R group. As markers of myocardial necrosis, the serum levels of cardiac enzyme CK-MB and cTnT were highly increased in I/R group and MF-1 group, as compared to PlGF group (P<0.05). It is worth mentioning that, the MF-1 group hearts exhibited more severe myocardial damage as compared to the I/R group. This observation was consistent to our previous findings that the MF-1 group hearts had larger infarct size and lower cardiac functional recovery. (Figs 2 and 3)

### PlGF ameliorated myocardial apoptosis after I/R

**Fig 4:**
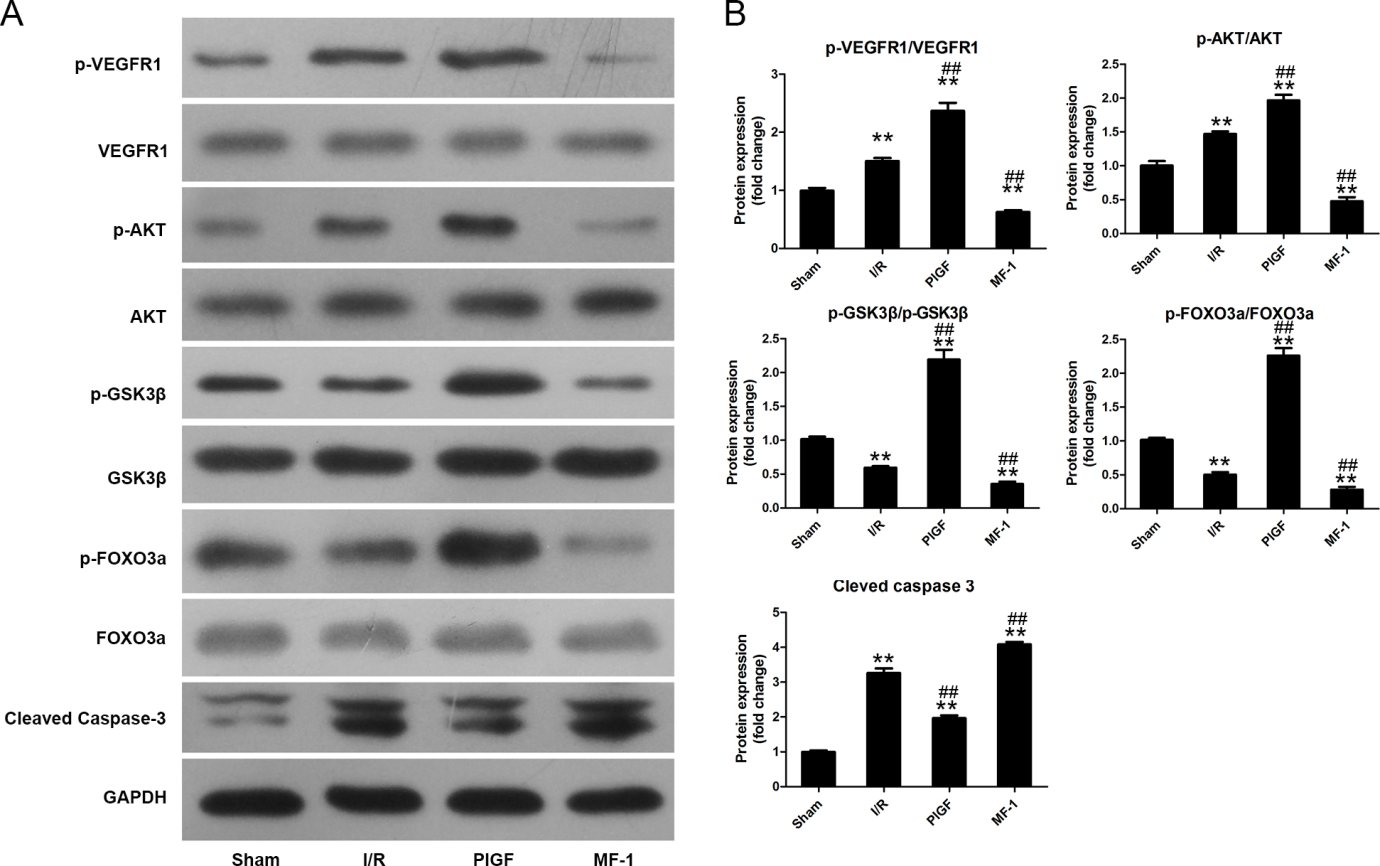
The effect of PlGF treatment on the activation of AKT, GSK-3β, FoxO3a, and caspase 3. (A) and (B) The effect PlGF pretreatment on on the activation of AKT, GSK-3β, FoxO3a, and caspase 3 (n = 6). Data were expressed as mean ± SEM. ** *P*<0.01 vs. the Sham group, # *P*<0.05 vs. the I/R group, ## *P*<0.01 vs. the I/R group.

**Fig 5:**
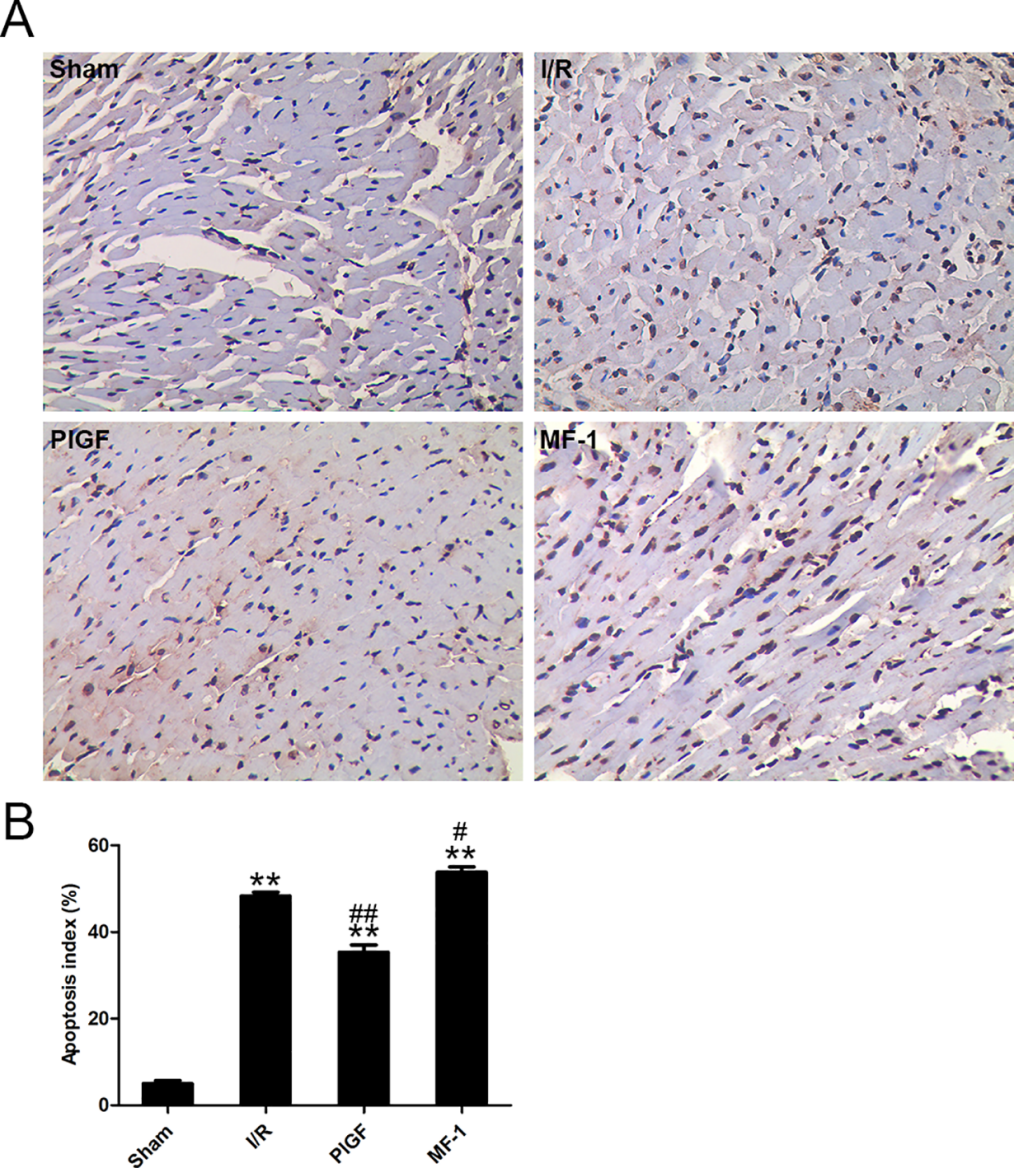
The effect of PlGF treatment on myocardium apoptosis. (A) and (B) show the representative images of the apoptotic cardiomyocytes after reperfusion (n = 6). Data were expressed as mean ± SEM. ** *P*<0.01 vs. the Sham group, # *P*<0.05 vs. the I/R group, ## *P*<0.01 vs. the I/R group.

**Fig 6:**
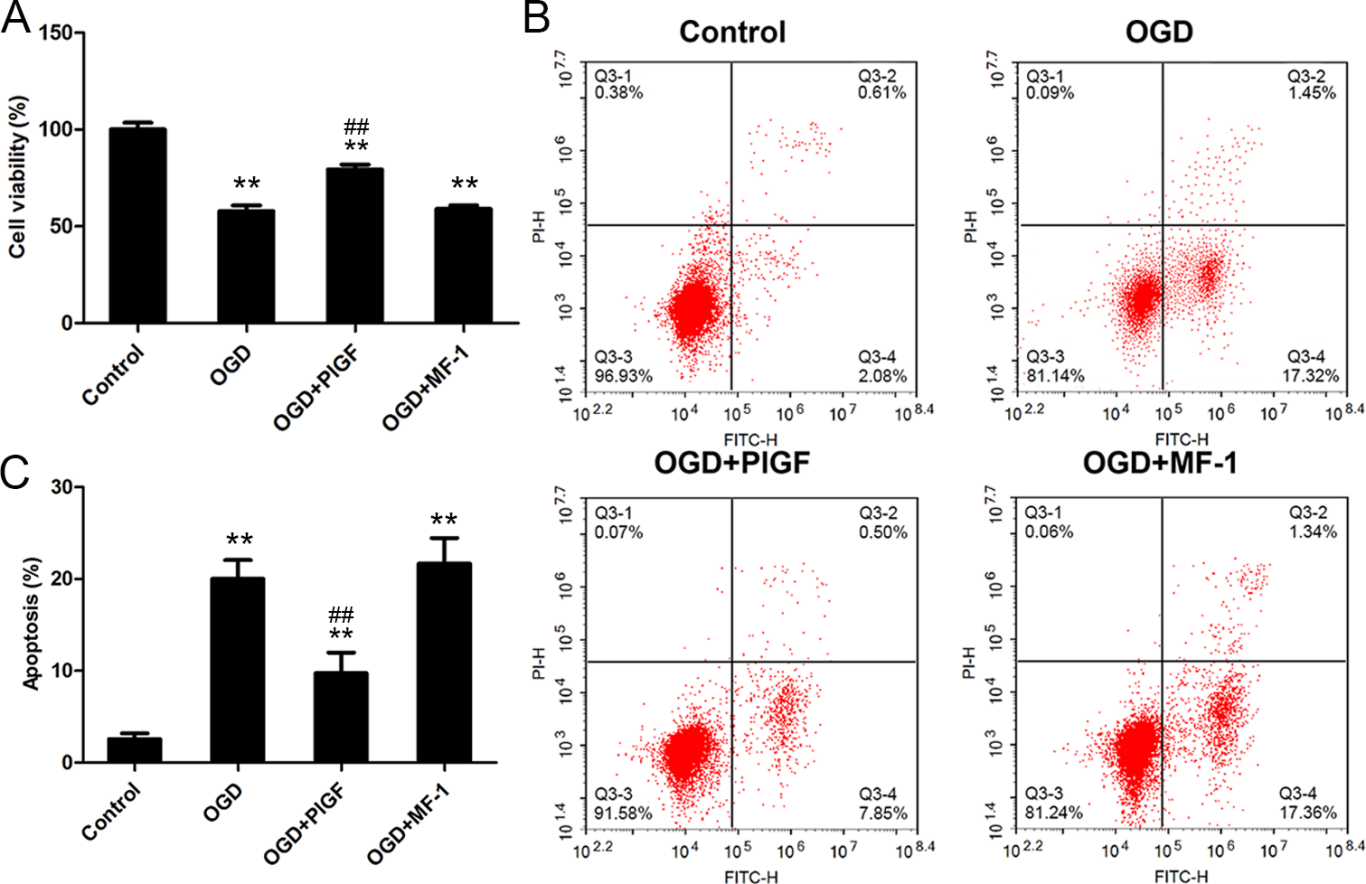
The effect of PlGF on cell viability and apoptosis of cardiomyocytes. (A) shows cell viability of cardiomyocytes after reoxygenation (n = 4). (B) and (C) show apoptosis of cardiomyocytes after reoxygenation (n = 4). Data were expressed as mean ± SEM. ** *P*<0.01 vs. the Control group, ## *P*<0.01 vs. the OGD group. OGD: Oxygen Glucose Deprivation.

As shown in Fig 4, pretreatment with PlGF significantly increased VEGFR1 phosphorylation (vs. the I/R group, P<0.01). Through VEGFR1 phosphorylation, PlGF significantly increased the phosphorylation of AKT and subsequently, increased the phosphorylation of GSK3β and FoxO3a (vs. the I/R group, P<0.01). PlGF significantly decreased Caspase-3 cleavage as well (vs. the I/R group, P<0.01). In contrast, treatment with MF-1 conferred the opposite effect, decreasing the phosphorylation of VEGFR1 (vs. the I/R group, P<0.01) and the downstream targets AKT, GSK3β and FoxO3a. The protection provided by PlGF and the opposite effect from MF-1 were also demonstrated by TUNEL staining. (Figs 5 and **6**)

### PlGF attenuated cardiomyocyte apoptosis and oxidative stress induced by OGD

Compared with OGD and OGD+PLGF+MF-1 groups, activation of VEGFR1 with PlGF significantly improved cell viability after H/R (Fig 5A). The cell apoptosis induced by H/R was also largely prevented by PlGF (9.6±2.7%) as compared with the OGD group (19.6.5±2.4%, P<0.01), while this protection was totally inhibited by MF-1 (the OGD+PLGF+MF-1 group, 21.7±3.3%, P<0.01). (Fig 6B and 6C)

**Fig 7:**
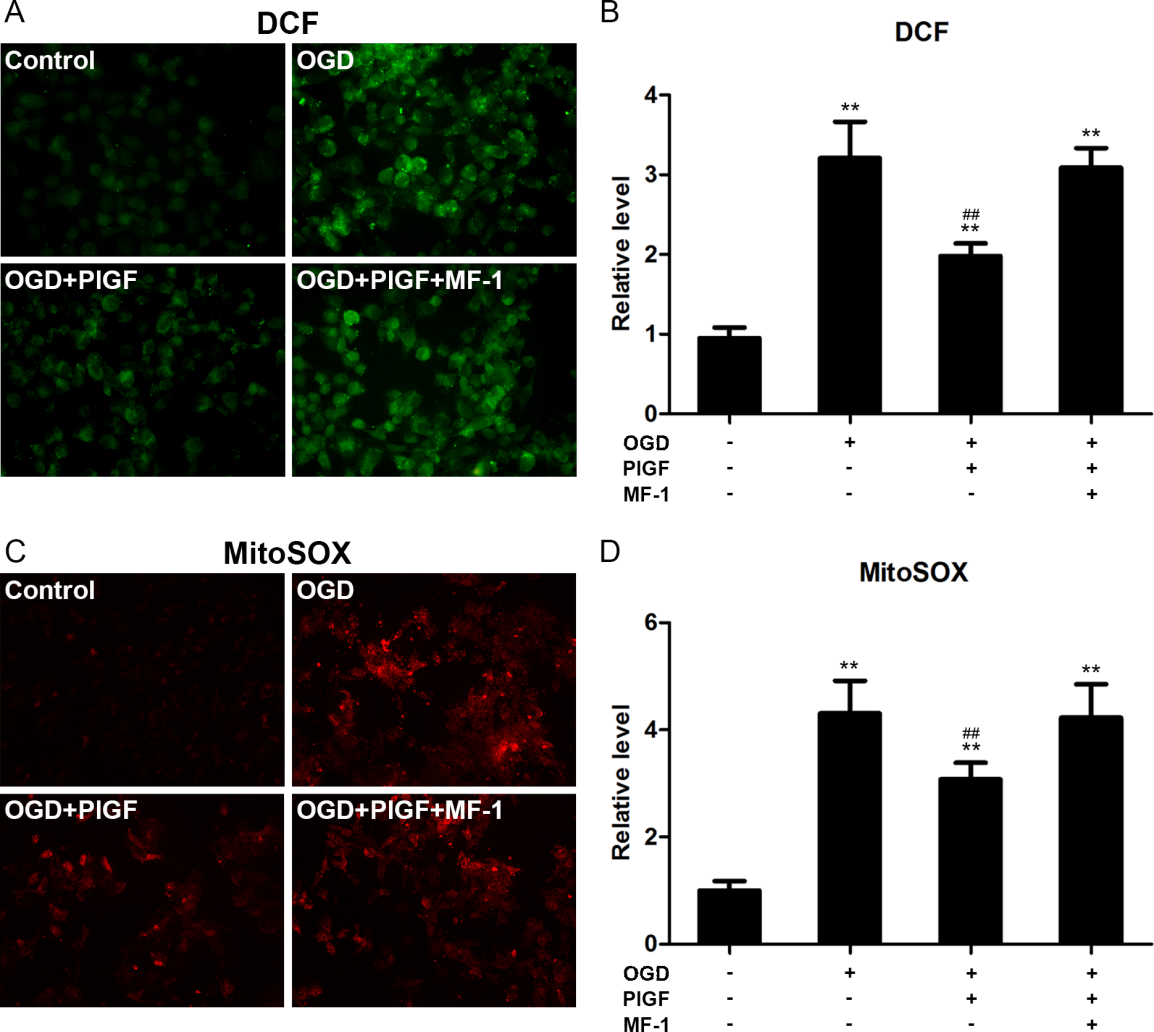
The effect of PlGF on reactive oxygen species (ROS) after hypoxia/reoxygenation. (A) and (B) show intracellular superoxide detected by DCF after reoxygenation (n = 4). (C) and (D) show mitochondrial superoxide detected by MitoSOX after reoxygenation (n = 4). Data were expressed as mean ± SEM. ** *P*<0.01 vs. the Control group, ## *P*<0.01 vs. the OGD group. DCF: 20,70-dichlorofluorescein diacetate, MF-1: neutralizing monoclonal Flt-1 antibody, OGD: Oxygen Glucose Deprivation.

After H/R, the intracellular superoxide detected by DCF was greatly increased in the OGD and OGD+PLGF+MF-1 groups (P<0.01, vs. the OGD group) but significantly reduced in the PlGF group (P<0.01 and P<0.01 vs. the OGD and OGD+PLGF+MF-1 groups, respectively, Fig 7). Consistently, the fluorescence of MitoSOX was suppressed in the PlGF group in comparison with the OGD and OGD+PLGF+MF-1 groups.

### Discussion

Early myocardial reperfusion is the most effective strategy for treating AMI and improving the clinical outcome. However, the beneficial effects of myocardial reperfusion could be reduced by MIRI. It may partly explain why the rate of death approaches 10% and the post-incidence of cardiac failure is almost 25% after AMI, despite optimal myocardial reperfusion. Results from animal models of AMI show that MIRI contributes for up to 50% of the final size of the myocardial infarction. In this study, we demonstrated that the activation of VEGFR1 by PlGF played a pivotal role in ameliorating MIRI.

Earlier experimental studies have shown that the activation of VEGFR1 by PlGF could improve cardiac function and survival rate in animal models experiencing heart failure by enhancing angiogenesis and arteriogenesis.[10, 23–25] The angiogenic growth factor, PlGF, was also reported to be involved in the growth and spread of cancer and that PlGF and Flt-1 were expressed in 36% to 60% and 65% of primary breast cancers respectively, which suggested that PlGF may also be active in cell growth and metastasis.[26] In addition, the administration of PlGF after AMI induced not only enlargement of vessel size, but also compensatory hypertrophy of cardiomyocyte in remote non-infarcted myocardium.[13, 23, 27] As such, PlGF may have direct protective effects on the myocytes against MIRI other than the effect of angiogenesis.

The anti-apoptotic and non-angiogenic properties of VEGFR1 activation have been confirmed. In a recent study, PlGF and VEGFB, selective ligands of VEGFR1, exerted powerful antiapoptotic effect in both cultured cardiomyocytes and after myocardial infarction in vivo.[28] The prolonged intramyocardial expression of VEGFB on adeno-associated virus-mediated gene significantly improved cardiac function after myocardial infarction and prevented loss of cardiac mass in the absence of angiogenesis. In present study, VEGFR1 activation prevented the cultured cardiomyocytes from apoptosis induced by hypoxia and oxidative stress.

Many studies have shown the cardioprotection of ischemic preconditioning via various signaling pathways.[7, 29–34] Studies demonstrated that the heterozygous knockout of VEGFR1 reduced cardioprotection of ischemic preconditioning.[15, 16] VEGFR1 heterozygous knockout mouse hearts are more sensitive to reperfusion injury, which suggests the involvement of VEGFR1 in cardioprotection. Here we reported that PlGF pretreatment attenuated MIRI. GSK-3 and FoxO3a are 2 major targets of Akt which could also be activated by VEGFR1. The phosphorylation of GSK3β has been shown to protect against organ ischemic injury, oxidative stress, and apoptosis by enhancing Nrf2 expression which plays a critical role in the defense against oxidative stress.[35, 36] The activation of Akt/FoxO3a has been reported to improve mitochondrial function after MIRI.[37] The cleaved caspase-3 is the terminal common effector of the apoptotic pathway, and the activation of Akt is a major negative regulator of apoptosis.[22, 38] We found PlGF treatment enhanced the phosphorylation of Akt, GSK-3 and FoxO3a and inhibited the activation of caspase-3 after reperfusion. Consequently, the apoptosis index was significantly reduced by PlGF in vivo. Consistent with this, cell viability of the cultured cardiomyocytes were significantly enhanced with the treatment of PlGF under the condition of H/R.

In previous studies, PlGF was shown to be related to recruitment and chemotaxis in monocytes after ligation of the femoral artery.[39, 40] Studies reported that PlGF induced cardiac fibroblasts to secrete chemotactic cytokine such as TNF-α, IL6, IL1β, and Cxcl1.[24, 39] In addition, up-regulation of PlGF was reported to play a proinflammatory role in allergic asthma via increasing tissue neutrophilia and IL-17 production.[41] In our treatment strategy, the mouse hearts treated with PlGF showed reduced inflammation after reperfusion. This may indirectly result from the cardioprotective effects of PlGF resulting in less severe MIRI.

MF-1, neutralizing monoclonal antibody for VEGFR1/Flt-1, could block the interaction of ligands with VEGFR1, subsequently suppressing tumor cell proliferation and angiogenesis, and leading to tumor cell apoptosis.[42, 43] In this study, MF-1 treatment reversed the anti-oxidant effects of PlGF in cultured cardiomyocytes. We found MF-1 treatment aggravated the MIRI, which suggested that the endogenous VEGFR1 signaling axis was also blocked.

During reperfusion, with the return of oxygen, ROS formation has been shown to occur and increase significantly. ROS has been considered to be of paramount importance for MIRI development as extensive oxidative stress caused by ROS results in loss of cell viability, inflammation and cell death.[44] The elevated ROS is the primary activator of the mitochondrial permeability transition pore (mPTP). The opening of the mPTP would lead to mitochondrial depolarization, swelling, and ultimately apoptotic cell death.[44, 45] Reducing ROS has been shown to reduce MIRI.[46] It has been reported that VEGFR1 activation can reduce oxidative stress in cultured neonatal cardiomyocytes exposed to angiotensin II.[21] In present study, we found the selective VEGFR1 ligands PlGF prevented mitochondrial and cytosolic superoxide in cultured cardiomyocytes exposed to OGD (Fig 7). Our results suggested that the cardioprotection of PIGF in vitro may come from the anti-oxidant effects, and we inferred that the cardioprotection mechanism was the same of PIGF in vivo.

In summary, the present study demonstrated that pretreatment with PlGF could attenuate MIRI, leading to improved cardiac function. The activation of VEGFR1 with PlGF proved to be beneficial in inhibiting oxidative stress and reducing apoptosis. This may ultimately provide a superior therapy for patients suffering from cardiac I/R injury.
